# The Value of the Sequential Organ Failure Assessment (SOFA) Score and Serum Lactate Level in Sepsis and Its Use in Predicting Mortality

**DOI:** 10.7759/cureus.42683

**Published:** 2023-07-30

**Authors:** Sulakshana Sekhar, Vinay Pratap, Kumar Gaurav, Samir Toppo, Anil K Kamal, Rahul Nair, Eesha Ashok, Praveenkumar A

**Affiliations:** 1 General Surgery, Rajendra Institute of Medical Sciences (RIMS), Ranchi, IND; 2 Surgery, Rajendra Institute of Medical Sciences (RIMS), Ranchi, IND; 3 Internal Medicine, Rajendra Institute of Medical Sciences (RIMS), Ranchi, IND; 4 Surgery, Srirama Chandra Bhanja (SCB) Medical College and Hospital, Cuttack, IND

**Keywords:** predictor, correlation, mortality, serum lactate, sequential organ failure assessment (sofa), sepsis

## Abstract

Background and objective

Sepsis is a major health burden that leads to significant morbidity and mortality. Early diagnosis and severity prediction using various scoring systems can reduce the mortality rate, particularly in developing nations. There are two aims of this study. One is to evaluate the prognostic accuracy of the Sequential Organ Failure Assessment (SOFA) score and serum lactate levels in patients with sepsis to predict mortality. The other aim is to evaluate the relationship between the SOFA score and lactate so that we may be able to use lactate as a surrogate predictor of organ dysfunction and mortality in sepsis.

Methods

An observational prognostic accuracy study was conducted in the Department of General Surgery, Intensive Care Unit (ICU), Rajendra Institute of Medical Sciences (RIMS), Ranchi, Jharkhand, India, between 1^ ^July 2021 and 1 October 2022. We selected 128 patients, calculated their SOFA and lactate levels, and divided them into survivors and non-survivors according to their outcomes after seven days of assessment. The SOFA score and serum lactate levels were assessed as predictors of mortality, and their correlation was studied.

Results

We observed a significant decreasing trend in the value of the mean SOFA, maximum SOFA, mean lactate, and maximum lactate among survivors, whereas an increasing trend for the same was observed in non-survivors. The receiver operating characteristic (ROC) analysis showed the best diagnostic accuracy of the mean lactate (area under the curve {AUC}=0.996, 95% confidence interval {CI}=0.964-1.00, p≤0.0001). The maximum lactate (AUC=0.987, 95% CI=0.949-0.999, p≤0.0001) and mean SOFA scores (AUC=0.986, 95% CI=0.948-0.999, p≤0.0001) were good at predicting the mortality in sepsis. A slightly lower diagnostic accuracy was found for the maximum SOFA score (AUC=0.969, 95% CI=0.923-0.992, p≤0.0001). There was a strong correlation between the mean lactate and the mean SOFA with a correlation coefficient of 0.883 and p=0.0001. A good correlation was found between maximum lactate and maximum SOFA too (correlation coefficient=0.873, p≤0.0001).

Conclusion

This study highlights the different predictors of mortality in the patients with sepsis. The maximum lactate was the most accurate in predicting mortality in sepsis. It also demonstrates how serum lactate, due to its strong correlation with the SOFA score, can be used in its place to predict mortality in sepsis and organ dysfunction.

## Introduction

Sepsis is a significant global health problem, with 48.9 million cases and 11 million sepsis-related deaths reported worldwide in the year 2017 [1]. Incidence and mortality rates vary worldwide, with the highest burden found in developing nations in sub-Saharan Africa, Oceania, and Asia. The early diagnosis and treatment of sepsis are crucial due to its high mortality rate.

The Third International Consensus Definitions Task Force (SEPSIS 3) defined sepsis as life-threatening organ dysfunction caused by a dysregulated host response to infection [2]. The Task Force redefined systemic inflammatory response syndrome (SIRS) as a nonspecific organ dysfunction resulting from a dysregulated host response to infection [3]. The Task Force removed SIRS as a means of defining sepsis due to its inadequate specificity and sensitivity [4]. The SIRS criteria do not necessarily indicate a dysregulated, life-threatening response and are present in many hospitalized patients, including those who never develop infection and never incur adverse outcomes (poor discriminant validity) [5]. SIRS is diagnosed by the fulfillment of any two of the following criteria: body temperature of >38 degrees Celsius or <36 degrees Celsius, heart rate of >90 beats per minute, respiratory rate >20 cycles/minute or partial pressure of CO_2_ of <32 millimeters of mercury (mmHg), and white blood cell count of >12000/cubic millimeter (mm^3^) or <4000/mm^3^ or >10% band or immature forms.

The Sequential Organ Failure Assessment (SOFA) Score or bedside quick SOFA (qSOFA) is the new tool for measuring organ dysfunction according to SEPSIS 3. SOFA grades the function of six organ systems, including the central nervous system, cardiovascular system, respiration, coagulation, liver, and kidney, on a scale of 0-4 based on the degree of dysfunction using objective measurements [6]. SOFA has demonstrated good predictive power in determining mortality in sepsis patients [7]. Vincent et al. [8] and Moreno et al. [9] proved through their studies that SOFA and its sub-scores can help in predicting mortality in sepsis.

Traditionally, hyperlactatemia in sepsis has been attributed to anaerobic glycolysis and decreased oxygen delivery to tissues, indicating hypoperfusion of tissues. The increased stimulation of beta-adrenergic receptors by epinephrine causes increased glycolysis and pyruvate production, overwhelming the tricarboxylic acid (TCA) cycle. Some of the pyruvates get converted to lactate, resulting in hyperlactatemia [10]. Lactate is also a marker of endogenous catecholamine release, which can help detect patients with occult shock who are maintaining blood pressure only due to endogenous catecholamines.

The Surviving Sepsis Guidelines of 2012 emphasized the importance of early screening and detection of severe sepsis. It recommended the routine use of screening tools in seriously ill patients to decrease mortality. Measuring lactate levels in the patients suspected of being gravely ill helps in the quicker diagnosis and initiation of treatment. This recommendation was made after research was done on the improvement in mortality when occult tissue hypoperfusion is identified and treated early [11].

With the new definitions and management strategies of sepsis under SEPSIS 3, the early management of sepsis and the prevention of multiorgan dysfunction, which is the root cause of sepsis mortality, are crucial. SOFA can be used to evaluate organ dysfunction and assess the severity of sepsis and predict its outcomes. Serum lactate is an indicator of hypoperfusion or increased stimulation of adrenergic receptors and can help predict outcome in sepsis patients.

There have been multiple studies and researches done on the predictors of sepsis in the developed nations. There is a lack of data using the same predictors in a practical sense in the hospitals of India. Applying these predictors of mortality to a new population, we wanted to see which of them would emerge as the most accurate.

There were two aims of this study. One was to evaluate the prognostic accuracy of the SOFA score and lactate in predicting mortality in sepsis. The other was to assess the association between the SOFA score and lactate level. This study throws light on mortality prediction in prognosticating sepsis in a tertiary care center in Ranchi, Jharkhand, where no previous record of sepsis prognostication exists. There is a dearth of studies, especially from this region of India, depicting the relationship between the SOFA score and serum lactate, which is a surrogate or indirect marker of mortality in sepsis. A novel study in Jharkhand demonstrating the tools to predict mortality in sepsis can help in the early diagnosis, prognostication, and better management of the disease.

## Materials and methods

Study design and sampling

This was a prognostic accuracy model study conducted over a period of 16 months in the Department of General Surgery at Rajendra Institute of Medical Sciences (RIMS), Ranchi. The study was approved by the Institutional Ethics Committee of Rajendra Institute of Medical Sciences, Ranchi (MEMO NO-265, dated 19 June 2021). The inclusion and exclusion criteria are discussed below (Table 1).

**Table 1:** Inclusion and exclusion criteria. RIMS, Rajendra Institute of Medical Sciences; SIRS, systemic inflammatory response syndrome

Inclusion criteria	Exclusion criteria
Patients who were admitted to the Department of General Surgery, Intensive Care Unit, RIMS, Ranchi	Patients with comorbidities such as diabetes mellitus, known hypertensive or heart disease, and cancer
Admitted between 1 July 2021 and 1 October 2022	Those with a hospital stay of fewer than 48 hours
Age over 15 years	
Two or more SIRS criteria were positive	

The sample size was initially determined using the following formula: SS=z2 p(1-p)/e^2 ^(SS=sample size, z=statistic for a level of confidence, p=proportion, and e=margin of error). As z=1.96 (taking a confidence interval (CI) of 95%), p=0.5 (there is no published data regarding the prevalence of sepsis in the state of Jharkhand, so it has to be assumed to be 50%), and e=0.05, then SS=384. Taking the formula for finite population size (N), the new sample size (SS’) was determined using the following formula: SS’=SS/(1+SS/N). Here, N=192, the number of patients admitted with the diagnosis of sepsis in RIMS, Ranchi, in the years 2020-2021. So, SS’=128.

The study protocol was explained to the patients who fulfilled the inclusion criteria, and informed consent was obtained, with confidentiality guaranteed. The SOFA score was calculated every day for a period of seven days for all the patients being evaluated. Arterial blood gas analysis was done daily to calculate the lactate level. The mean and maximum values of SOFA and lactate were calculated for each patient. The SOFA and serum lactate levels were assessed until discharge from surgical intensive care unit (SICU) or death (not more than seven days). On analyzing the data retrospectively, the patients were divided into two groups, survivors and non-survivors, based on the outcome. The characteristics, SOFA score, and serum lactate level between the two groups were compared. The correlation between mortality and the SOFA score and mortality and serum lactate level was also evaluated.

Operational definitions

Sepsis

In this study, any patient fulfilling at least two of the SIRS criteria is considered to have sepsis.

Mean SOFA

The mean SOFA is the average of the SOFA values for a particular patient, day, or group.

Maximum SOFA

The maximum SOFA is the highest SOFA value of a patient, day, or group.

Mean Lactate

The mean lactate is the average serum lactate values for a particular patient, day, or group.

Maximum Lactate

The maximum lactate is the highest serum lactate value for a patient, day, or group.

Statistical analysis

Categorical variables were presented as numbers and percentages. They were name, age group, sex, and mortality. On the other hand, quantitative data such as age, mean lactate, maximum lactate, mean SOFA, and maximum SOFA were presented as the mean±standard deviation (SD) and as median with 25th and 75th percentiles (interquartile range).

The association of the variables, which were quantitative in nature, was analyzed using the independent t-test. The association of qualitative variables was analyzed using the chi-square test. The receiver operating characteristic (ROC) curve was used to find the positive predictive value and negative predictive value of SOFA and lactate for predicting mortality.

The Pearson correlation coefficient was used for the correlation between lactate and the SOFA score. The data entry was done in the Microsoft Excel (Microsoft® Corp., Redmond, WA) spreadsheet, and the final analysis was done with the use of the Statistical Package for Social Sciences (SPSS) version 25.0 (IBM SPSS Statistics, Armonk, NY). A p-value of less than 0.05 was considered statistically significant for all.

## Results

The mean age of the patients in this study was 41.68±17.1 years. The most common age group affected was 21-30 years (28.91%), and the least common age group was >70 years (4.69%). The patients’ ages ranged from 16 to 85 years (Table 2).

**Table 2:** Age distribution of the patients in the study.

Age in years	Frequency	Percentage
16-20	8	6.25%
21-30	37	28.91%
31-40	26	20.31%
41-50	17	13.28%
51-60	18	14.06%
61-70	16	12.50%
>70	6	4.69%

Eighty-three patients (64.84%) were male, and 45 patients (35.16%) were female. The sex ratio was 1.84:1 (male/female) (Table 3).

**Table 3:** Gender distribution among the patients.

	Number	Percentage
Male	83	64.84%
Female	45	35.16%

In this study, 99 out of a total of 128 patients (77.34%) survived, while 29 (22.66%) died. The mean SOFA score in the survivor group was 2.39±1.54 and 7.35±1.38 in the non-survivor group. The p-value, which was calculated by using the independent t-test, was <0.0001. Therefore, the results demonstrating higher mean SOFA scores in the non-survivor group were statistically significant. The mean lactate in the non-survivor group was 7.36±1.82 and 2.25±0.89 in survivors group (Table 4).

**Table 4:** Characteristics of survivors and non-survivors. SOFA: Sequential Organ Failure Assessment

	Number	Percentage	Mean SOFA	Mean lactate
Survivors	99	77.34%	2.39	2.25
Non-survivors	29	22.66%	7.35	7.26

In the survivor group, the mean SOFA score on day 0 was 3.65±1.79, and it decreased to 1.05±1.27. However, in the non-survivor group, the mean SOFA score on day 0 was 6.41±1.68, which increased to 8±0.82 on day 6. On day 7, all patients in the non-survivor group had died. The difference in the mean SOFA score between day 0 and day 7 showed a decrease in the survivor group and an increasing trend in the non-survivors (Table 5).

**Table 5:** Day-wise trend of the mean SOFA and mean lactate. SOFA, Sequential Organ Failure Assessment; mmol/L, millimole per liter; SD, standard deviation

SOFA score	Mean±SD	Lactate (mmol/L)	Mean±SD
At day 0	4.27±2.11	At day 0	3.94±2.13
At day 1	4.33±2.25	At day 1	3.78±2.15
At day 2	3.83±2.28	At day 2	3.51±2.18
At day 3	3.35±2.35	At day 3	3.16±2
At day 4	3.05±2.48	At day 4	2.96±2.09
At day 5	2.51±2.57	At day 5	2.54±1.98
At day 6	1.84±2.18	At day 6	2.02±1.57
At day 7	1.05±1.27	At day 7	1.39±0.6

In the survivor group, the mean lactate on day 0 was 3.12±1.19, and it decreased to 1.39±0.64. However, in the non-survivor group, the mean lactate on day 0 was 6.73±2.25, which increased to 7.21±1.11 on day 6 (Table 4). The difference in the mean lactate between day 0 and day 7 showed a decrease in the survivor group and an increasing trend in the non-survivors.

Day-wise SOFA score calculated for survivors and non-survivors has been shown in Table 6.

**Table 6:** Association of the SOFA score with outcome in survivors and non-survivors. ^‡^An independent t-test was used to find the p-value SOFA, Sequential Organ Failure Assessment; SD, standard deviation

SOFA score	Non-survivors	Survivors	Total	P-value
At day 0				
Mean±SD	6.41±1.68	3.65±1.79	4.27±2.11	<0.0001^‡^
Median (25th-75th percentile)	6 (5-8)	3 (2-4)	4 (3-6)	
Range	4-9	1-9	1-9	
At day 1				
Mean±SD	6.9±1.68	3.58±1.8	4.33±2.25	<0.0001^‡^
Median (25th-75th percentile)	6 (6-8)	3 (2-4)	4 (2-6)	
Range	5-10	1-9	1-10	
At day 2				
Mean±SD	7.04±1.51	3.02±1.64	3.83±2.28	<0.0001^‡^
Median (25th-75th percentile)	7 (6-8)	3 (2-4)	3 (2-6)	
Range	5-10	0-8	0-10	
At day 3				
Mean±SD	7.05±1.33	2.53±1.62	3.35±2.35	<0.0001^‡^
Median (25th-75th percentile)	7 (6-8)	2 (1-3)	3 (1-5)	
Range	5-10	0-7	0-10	
At day 4				
Mean±SD	7.35±1.14	2.18±1.62	3.05±2.48	<0.0001^‡^
Median (25th-75th percentile)	7 (7-8)	2 (1-3)	2 (1-4.5)	
Range	6-10	0-7	0-10	
At day 5				
Mean±SD	7.93±0.88	1.69±1.52	2.51±2.57	<0.0001^‡^
Median (25th-75th percentile)	8 (7-8)	1 (1-2)	2 (1-4)	
Range	7-10	0-6	0-10	
At day 6				
Mean±SD	8±0.82	1.4±1.47	1.84±2.18	<0.0001^‡^
Median (25th-75th percentile)	8 (7.5-8.5)	1 (0-2)	1 (0-2.75)	
Range	7-9	0-7	0-9	
At day 7				
Mean±SD	-	1.05±1.27	1.05±1.27	NA
Median (25th-75th percentile)	-	1 (0-2)	1 (0-2)	
Range	-	0-6	0-6	

Day-wise lactate levels were analyzed for survivors and non-survivors (Table 7).

**Table 7:** Association of lactate with outcome in survivors and non-survivors. ‡An independent t-test was used to find the p-value SD, standard deviation; mmol/L, millimole per liter; NA, not available

Lactate (mmol/L)	Non-survivors	Survivors	Total	P-value
At day 0				
Mean±SD	6.73±2.25	3.12±1.19	3.94±2.13	<0.0001^‡^
Median (25th-75th percentile)	6.8 (4.7-8.7)	2.9 (2.3-3.7)	3.2 (2.5-4.625)	
Range	3.2-11	1.5-7.3	1.5-11	
At day 1				
Mean±SD	6.83±2.02	2.88±1.11	3.78±2.15	<0.0001^‡^
Median (25th-75th percentile)	7.1 (5.3-8.1)	2.7 (2.1-3.3)	3 (2.3-4.55)	
Range	3.7-10.9	1.4-7	1.4-10.9	
At day 2				
Mean±SD	6.98±1.89	2.6±1.03	3.51±2.18	<0.0001^‡^
Median (25th-75th percentile)	6.8 (5.375-8.05)	2.4 (2-3)	2.7 (2.1-4.1)	
Range	4.1-10.9	1.1-6.7	1.1-10.9	
At day 3				
Mean±SD	6.8±1.21	2.35±0.97	3.16±2	<0.0001^‡^
Median (25th-75th percentile)	6.6 (6.025-7.875)	2.1 (1.7-2.8)	2.3 (1.9-3.5)	
Range	4.6-8.8	0.9-6.5	0.9-8.8	
At day 4				
Mean±SD	7.08±1.3	2.13±0.88	2.96±2.09	<0.0001^‡^
Median (25th-75th percentile)	6.75 (6.15-8.25)	2 (1.55-2.5)	2.1 (1.65-3.05)	
Range	4.5-9.3	0.8-5.4	0.8-9.3	
At day 5				
Mean±SD	7.1±1.2	1.85±0.8	2.54±1.98	<0.0001^‡^
Median (25th-75th percentile)	6.8 (6.5-7.75)	1.7 (1.3-2.15)	1.8 (1.3-2.575)	
Range	5.2-9.1	0.6-4.7	0.6-9.1	
At day 6				
Mean±SD	7.21±1.11	1.65±0.71	2.02±1.57	<0.0001^‡^
Median (25th-75th percentile)	7.2 (6.4-7.95)	1.5 (1.2-2.05)	1.5 (1.2-2.1)	
Range	5.8-8.8	0.5-4.1	0.5-8.8	
At day 7				
Mean±SD	-	1.39±0.64	1.39±0.64	NA
Median (25th-75th percentile)	-	1.2 (1-1.7)	1.2 (1-1.7)	
Range	-	0.5-3.6	0.5-3.6	

**Figure 1:**
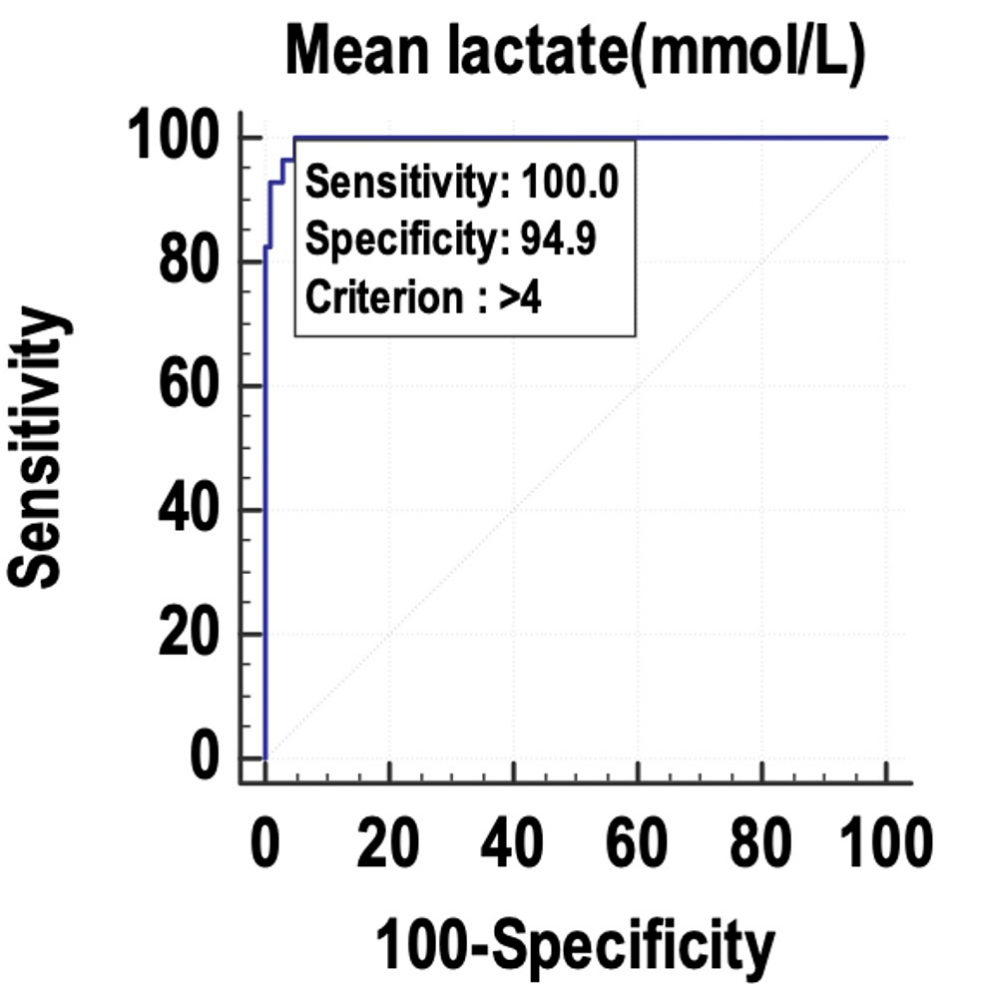
Receiver operating characteristic curve of the mean lactate as a predictor of mortality. mmol/L: millimole per liter

The mean SOFA, maximum SOFA, mean lactate, and maximum lactate were evaluated as predictors of mortality in the patients with sepsis. The mean lactate (Figure 1) was identified as the best predictor of mortality in sepsis, with an area under the receiver operating characteristic (AUROC) curve of 0.996 and 95% confidence interval (CI) of 0.964-1.

**Figure 2:**
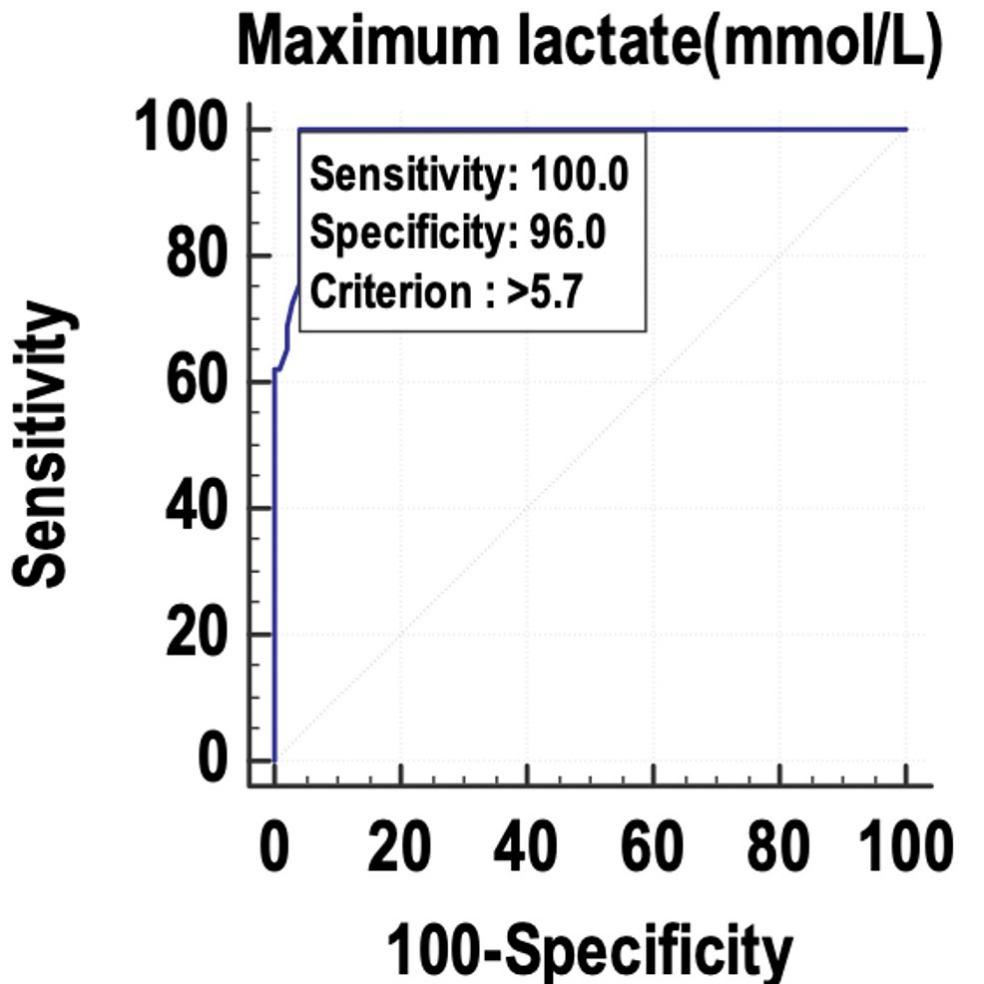
Receiver operating characteristic curve of the maximum lactate as a predictor of mortality. mmol/L: millimole per liter

**Figure 3:**
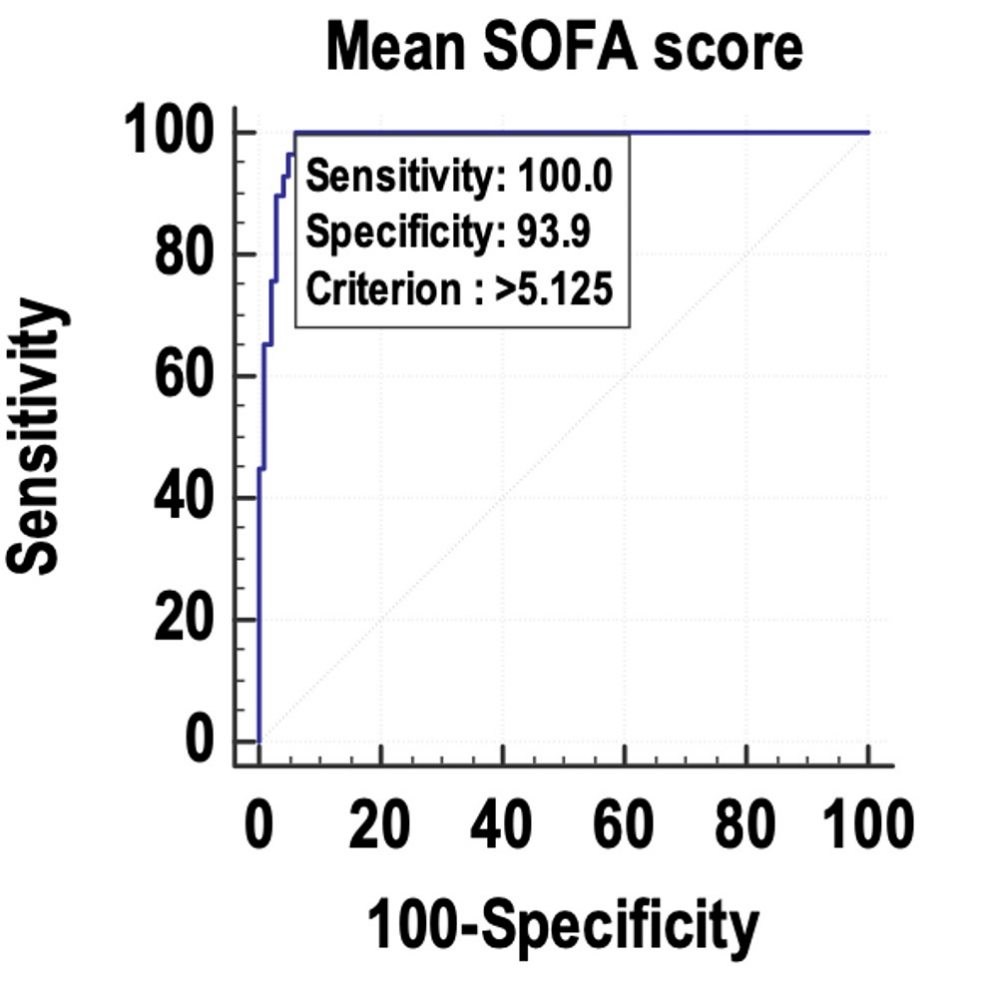
Receiver operating characteristic curve of the mean SOFA as a predictor of mortality. SOFA: Sequential Organ Failure Assessment

The maximum lactate (Figure 2) and the mean SOFA (Figure 3) were also found to be good indicators of mortality, with an AUROC of 0.987 and 0.986, respectively, and 95% CI of 0.94-0.99.

**Figure 4:**
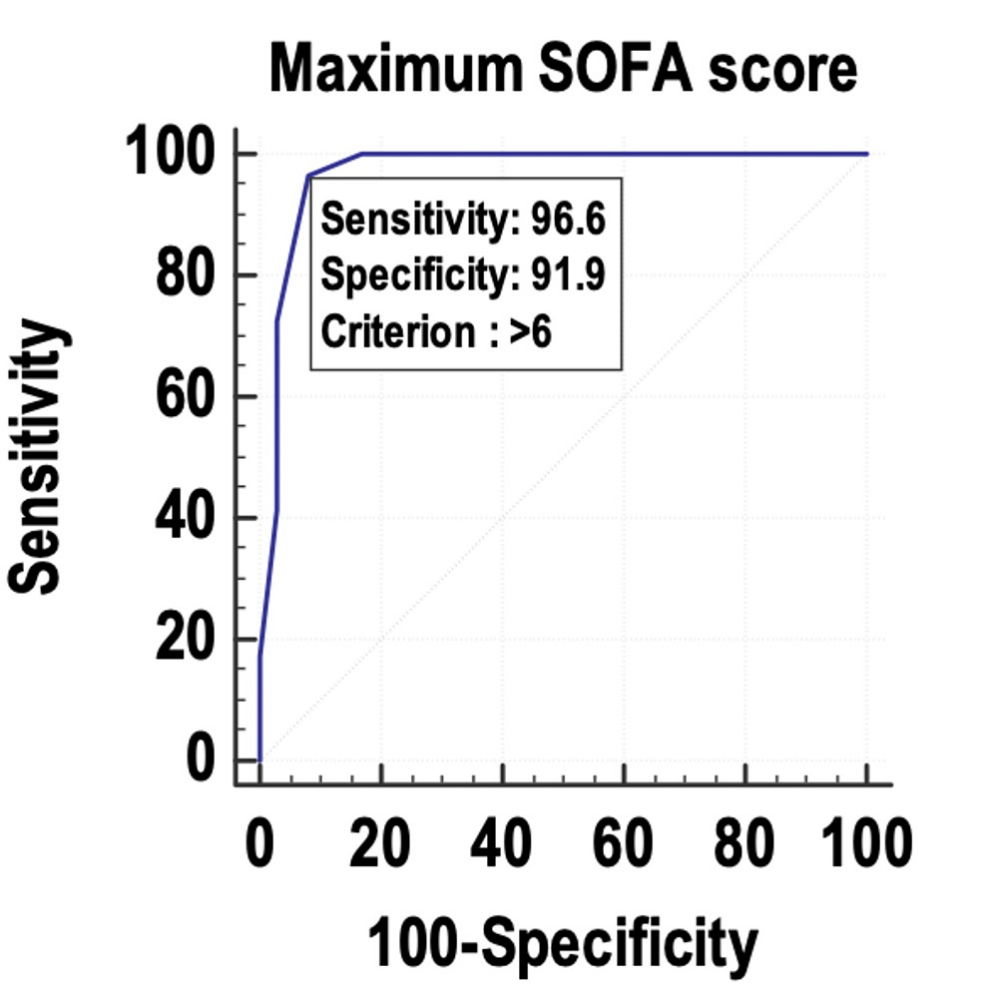
Receiver operating characteristic curve of the maximum SOFA as a predictor of mortality. SOFA: Sequential Organ Failure Assessment

The maximum SOFA (Figure 4) was the least accurate predictor among the four variables studied, with an AUROC of 0.969. However, all the four variables were independent predictors of mortality in sepsis.

**Figure 5:**
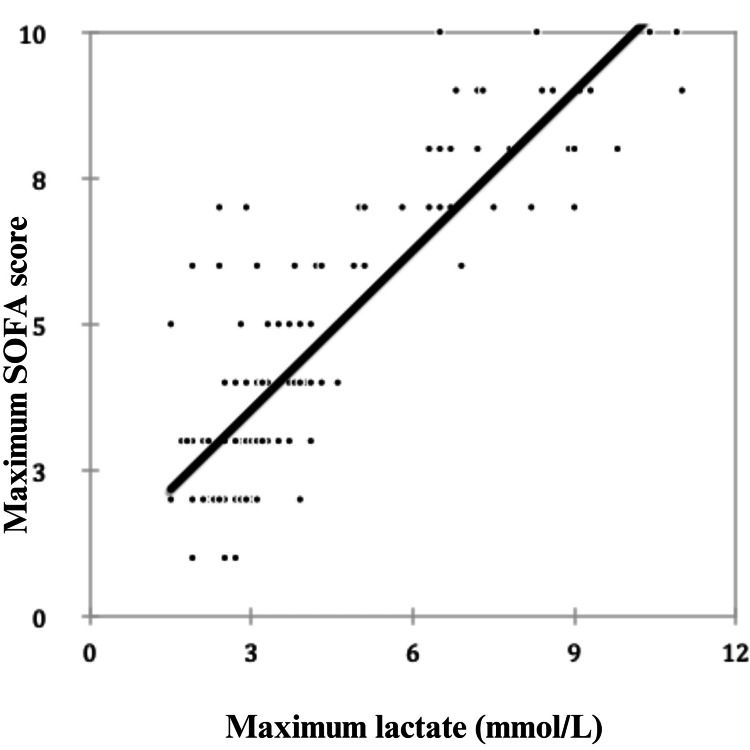
Scatter diagram showing the association between the maximum lactate and maximum SOFA. SOFA, Sequential Organ Failure Assessment; mmol/L, millimole per liter

The association between SOFA and lactate was assessed (Figure 5). The scatter diagram below shows the relationship between the maximum SOFA score and the maximum lactate level. The correlation coefficient is 0.873, which indicates a positive correlation between the maximum SOFA score and the maximum lactate level. There is a strong positive correlation between the two variables, indicating that a change in one variable causes variation in the same direction in the other variable.

**Figure 6:**
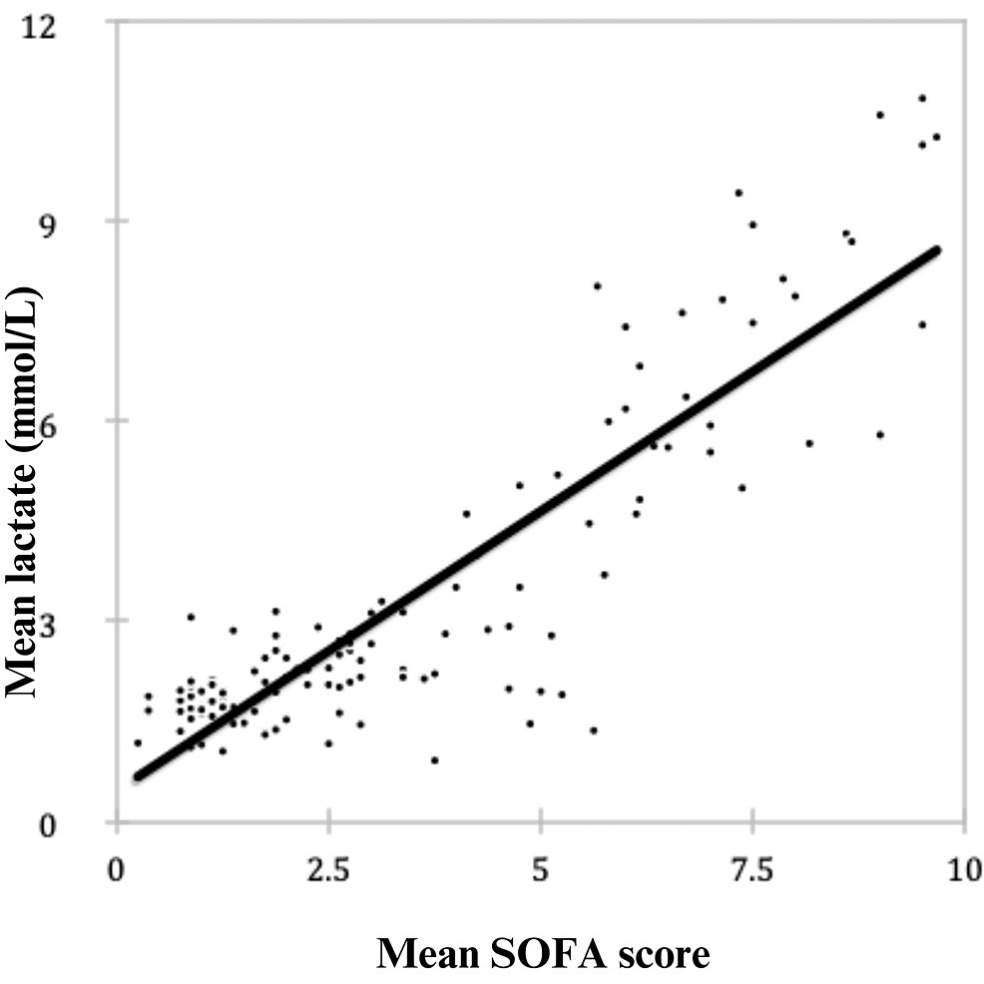
Scatter diagram showing the association between the mean SOFA and mean lactate. SOFA, Sequential Organ Failure Assessment; mmol/L, millimole per liter

The scatter plot below shows the relationship between the mean SOFA score and the mean lactate level (Figure 6). The correlation coefficient is 0.883, indicating a positive correlation between the mean SOFA score and the mean lactate level. There is a strong positive correlation between the two variables, indicating that a change in one variable causes variation in the same direction in the other variable.

## Discussion

This study conducted at Rajendra Institute of Medical Sciences, Ranchi, India, evaluated 128 patients with sepsis over 16 months, and the results were consistent with those from the rest of the world.

The mean age of the patients in this study was 41.68±17.1 years, and there was no clear correlation between age and mortality. Similar studies by Nasa et al. [12] reported that old age and very old age were independent risk factors and predictors of mortality, with the risk ratio of an elderly person succumbing to sepsis being >1. There was no significant relationship between the patient’s age and outcome. In their studies, Martin et al. [13] and Yang et al. [14] found that age was a significant and independent predictor of mortality.

In this study, 83 patients (64.84%) were male, and 45 patients (35.16%) were female. The sex ratio was 1.84:1 (male/female). Likewise, Nasir et al. [15] also concluded that males had a >70% mortality rate in sepsis. Out of a total of 128 patients, 99 patients (77.34%) survived, and 29 (22.66%) died. These mortality rates were comparable to those found in the studies of Hu et al. [16] who reported an 18.4% mortality rate of sepsis in the United States of America. However, the mortality rate in the study of Markwart et al. [17], conducted on hospital-acquired sepsis, was higher at 52.3%.

The mean SOFA score in survivors was 2.39±1.54, and in the non-survivor group, it was 7.35±1.38, which was statistically significant. The maximum SOFA score in survivors was 3.65±1.79, and in the non-survivor group, it was 8.28±1.13. The results depicting higher maximum SOFA scores in the non-survivor group compared to the survivor group were statistically significant. Multiple studies done in the past have compared various predictors of mortality in sepsis. The work of Ferreira et al. [18] showed that the mean and maximum SOFA scores were closely related to mortality, with the mean SOFA score having the strongest correlation with mortality, followed by the maximum SOFA. Similar results were obtained by Moreno et al. [9] who showed that the maximum SOFA and delta SOFA scores can be used to objectively measure organ failure in patients with sepsis in the intensive care unit (ICU). A multicenter study was carried out by Vincent et al. [8] in 1998, and they concluded that the SOFA score was a simple and useful clinical tool to predict organ failure in critically ill patients. They also advocated for its continued and regular use for a better understanding of mortality in sepsis. Corroborating these findings in their study, Jentzer et al. [19] indicated that there was an increasing trend of the mean SOFA in non-survivors and a decreasing trend in survivors, thus associating increasing SOFA score with mortality.

The mean lactate in survivors was significantly less than in the non-survivors. Similar results were obtained in the works of Villar et al. [20] and Meregalli et al. [21] who proved that elevated initial blood lactate levels are associated with a higher mortality rate. The incidence of postoperative complications was also higher in such patients.

The maximum SOFA, mean SOFA, maximum lactate, and mean lactate were used as predictors of mortality, and their sensitivity and specificity were calculated by plotting them on a receiver operating characteristic (ROC) curve. The area under the curve (AUC) was 0.996, and the diagnostic accuracy of predicting mortality was 96.09%. The mean lactate was found to have the best accuracy to predict mortality in the patients with sepsis. Other studies with similar findings include those by Liu et al. [22] and Ferreira et al. [18]. Liu et al. [22] studied the prognostic accuracy of the serum lactate, SOFA score, and qSOFA score in predicting mortality in sepsis. The area under the ROC curve was highest for the SOFA score (0.686) and was almost similar for serum lactate (0.664). The area under the curve of the ROC for qSOFA was 0.547. The authors concluded that serum lactate could be used as an independent predictor of mortality in sepsis, with similar accuracy to the SOFA score. Ferreira et al. [18] used the serial calculation and evaluation of SOFA scores in the patients with sepsis to predict mortality. The highest SOFA score had the largest area under the ROC curve (0.90) followed by the mean SOFA score (area under the ROC curve: 0.88). The authors concluded that the evaluation of the SOFA score throughout the ICU stay is a good prognostic indicator, especially the mean and the highest SOFA scores. An increase in the SOFA score during the first 48 hours of ICU admission, independent of the initial value, can predict mortality.

In this study, we have observed a very strong correlation between the mean and maximum serum lactate with the mean and maximum SOFA scores, indicating that lactate may also be used to indicate organ perfusion. Jansen et al. [23] corroborated the same results. In their study, they found a strong relationship between serum lactate and the SOFA score. This relationship was stronger during the early phase of stay in the ICU. However, these results are contrary to those obtained by Jemie et al. [24]. In their study, they found a poor and weak correlation between serum lactate levels and the 24-hour SOFA score and the 48-hour SOFA score (r=0.303 and r=0.449, respectively).

Although this study substantiates important data regarding sepsis, lactate, and SOFA, however, there were a few limitations; we could only include 128 patients. Being a single hospital-based study, a very small cohort of patients was selected. Hence, these results cannot be representative of the larger population and undermine the internal and external validity of the study. The absence of a longer follow-up period hid many secondary outcomes that may have occurred with time. The patients were evaluated for a short period of seven days only; thus, late causes of mortality were not accounted for.

## Conclusions

Sepsis and associated organ dysfunction are significant causes of death globally. We conducted this study in a tertiary care center in Ranchi, Jharkhand, with the aim of evaluating the prognostic accuracy of the SOFA score and serum lactate levels in patients with sepsis to predict mortality. The study concluded that out of the 77.34% of the patients that survived, the mean SOFA score was 2.39, and the mean lactate was 2.25. The most common age group affected was of 21-30 years of age. Non-survivors (22.66%) had a higher mean SOFA score of 7.35 and a mean lactate of 7.26. The representation of organ dysfunction using these values can help in the prognostication of the patients. Upon evaluating the different variables as predictors of mortality, it was found that the mean lactate was the best independent predictor with an AUROC of 0.996. There was a strong correlation between the mean lactate and mean SOFA (0.883) and between the maximum lactate and mean lactate (0.873). Lactate levels, due to their strong correlation with the SOFA score, can be used as a cost-effective and easy method to detect organ failure and help prioritize patients for intensive management. Systematic review and meta-analysis done on randomized controlled trials (RCTs) in sepsis show SOFA as a good predictor of mortality. However, there is a need for more studies and multivariate analysis to evaluate the efficacy of various predictors of mortality in sepsis. Using these findings, we may be able to save many more patients with limited resources and time.
